# Influence of Honey Bee Brood Protein on the Hydrophilic, Mechanical, and Thermal Properties of Polysaccharide Gel Films

**DOI:** 10.3390/gels11040236

**Published:** 2025-03-24

**Authors:** Suthaphat Kamthai, Pairote Wiriyacharee, Srisuwan Naruenartwongsakul, Patompong Khaw-on, Aree Deenu, Supakit Chaipoot, Rewat Phongphisutthinant, Kamonwan Tachai, Sawichaya Orpool

**Affiliations:** 1 Division of Packaging Technology, School of Agro-Industry, Faculty of Agro-Industry, Chiang Mai University, Chiang Mai 50100, Thailand; kamonwan.techai@gmail.com; 2 Lanna Rice Research Center, Chiang Mai University, Chiang Mai 50100, Thailand; 3 Faculty of Agro-Industry, Chiang Mai University, Chiang Mai 50100, Thailand; pairote.w@cmu.ac.th; 4 Center of Excellence in Microbial Diversity and Sustainable Utilization, Chiang Mai University, Chiang Mai 50200, Thailand; supakit.ch@cmu.ac.th (S.C.); rewat.p@cmu.ac.th (R.P.); 5 Processing and Product Development Factory, The Royal Project Foundation, Chiang Mai 50100, Thailand; 6 Division of Food Engineering, School of Agro-Industry, Faculty of Agro-Industry, Chiang Mai University, Chiang Mai 50100, Thailand; srisuwan.n@cmu.ac.th; 7 School of Nursing, Faculty of Nursing, Chiang Mai University, Chiang Mai 50200, Thailand; patompong.kh@cmu.ac.th; 8 Division of Food Science and Technology, School of Agro-Industry, Faculty of Agro-Industry, Chiang Mai University, Chiang Mai 50100, Thailand; aree_deenu@cmu.ac.th (A.D.); sawichayaop@gmail.com (S.O.); 9 Multidisciplinary Research Institute, Chiang Mai University, Chiang Mai 50200, Thailand

**Keywords:** honey bee brood, protein, polysaccharide, polysaccharide gel film

## Abstract

Growing concerns over the environmental impact of plastic packaging have driven interest in sustainable alternatives, particularly biopolymer-based films. This study developed ternary-blended polysaccharide gel films composed of carboxymethyl starch (CMS), chitosan (CS), and pectin (PT), with dialdehyde carboxymethyl cellulose (DCMC) as a crosslinker, and investigated the effects of honey bee brood protein (BBP) (0–0.4% *w*/*v*) on their mechanical, barrier, and thermal properties. A completely randomized design (CRD) was employed to evaluate the impact of BBP concentration on film characteristics. Results demonstrated that adding 0.4% BBP enhanced water vapor barrier properties and thermal stability while reducing hydrophilicity. The optimal formulation was observed at 0.1% BBP, providing the highest tensile strength (7.73 MPa), elongation at break (32.23%), and water-absorption capacity (369.01%). The improvements in thermal stability and hydrophilicity were attributed to BBP’s hydrophobic amino acids, which interacted with DCMC to form a denser polymer network, enhancing structural integrity and moisture resistance. Additionally, BBP incorporation contributed to the biodegradability of polysaccharide gel films, improving their environmental sustainability compared to conventional biopolymers. The findings suggest that BBP can serve as a functional additive in polysaccharide-based films, balancing performance and eco-friendliness for applications in biodegradable food and medical packaging.

## 1. Introduction

Recent research trends have facilitated the production of biopolymer films through the application of materials sourced from renewable and environmentally friendly origins as part of long-term global sustainable development goals. These advancements involve the use of natural biopolymers and the blending of different biopolymers to improve their properties, such as physical, mechanical, and barrier properties [1,2]. However, polysaccharide-based films such as starch and cellulose are hydrophilic and exhibit high swelling and gelatinization capacity when exposed to moisture, resulting in a poor water vapor barrier. These characteristics contribute to a reduction in the mechanical strength of polysaccharide biopolymer films and hinder their long-term stability, making them sensitive to moisture content [3,4,5]. However, the films prepared from polysaccharide biopolymer alone are not hydrophobic. The hydrophobicity and the high-water-resistance properties of polysaccharide-based films are due to the combination between nonpolar amino groups in natural proteins and functional additives.

To improve the polysaccharide-based gel films, the development of water-susceptibility properties, particularly, good water absorption, high thickness swelling, and low water solubility, together with an increase in the mechanical, gas barrier, and thermal properties can be increased using blends of different biopolymer-based substances, as well as physical or chemical treatment, along with the use of crosslinking agents. For instance, polysaccharides, proteins, and their derivatives may be used in different combinations to produce films with enhanced or tailored properties. These substances may be combined in different ways, including mixed, layered, or dispersed films [6]. In this case, pectin is capable of associating with other biopolymers such as chitosan, soy protein isolate, and cellulose-formed filmogenic solution for packaging film due to its anionic characteristic and good gelling properties [7]. An example of physical modification via electrostatic repulsion techniques could exceed electrostatic attraction in improving the tensile strength, oxygen barrier property, and thermal stability of a carboxymethyl starch/gelatin blended film [8]. Wang et al. [9] reported that gelatin/2,3-dialdehyde starch crystal films presented high tensile strength and enhanced hydrophobic properties, thermal stability, surface morphology, and so on because dialdehyde starch crystals can react and covalently crosslink with the amine groups of gelatin molecules.

Furthermore, several researchers have attempted to develop novel polysaccharide or protein-blended gel films, which can create a crosslinked polymeric chain holding significant amounts of water, absorbing in the polymer matrix and forming 3D structures by using different film constituents. To produce the extracellular polysaccharide gel film matrix, modified polysaccharides can be combined with crosslinking agents for crosslinking polymeric networks or chemical or physical contact, such as dialdehyde carboxymethyl cellulose (DCMC) and carboxymethyl starch (CMS) as crosslinking or compatibilizing agents. The addition of both chemicals during film preparation has led to better mechanical properties and thermal stability, low moisture sensitivity, and reduced hydrophilicity of biopolymer films, particularly protein-based films [10]. In such crosslinking reactions, the functional groups of crosslinking agents such as aldehydes [11] react effectively with amino groups numerously available in proteins and polysaccharide, especially for chitosan. For example, dialdehyde carboxymethyl cellulose (DCMC) showed that covalent crosslinking effectively increased the tensile strength of sericin films [12] and soy protein isolate films (up to 218%) [10].

Additionally, the Schiff base reaction between the amine group of the chitosan and the aldehyde group of DCMC has a great potential for generating gel formation and three-dimensional network structures through cross-chain reactions that establish crosslinking connections between biopolymer gel molecules, resulting in good mechanical strength and structural stability [13]. Another effect of this reaction on gel behavior indicated that the aldehyde group of DCMC and the amino group of dopamine-modified carboxymethyl chitosan (CS-DA) can act as a dynamic crosslink for controlling water absorption and swelling effects and endow the hydrogel with self-healing capability [14]. The aldehyde and polar groups in the DCMC structure enable the creation of hydrogen and covalent connections with proteins, including SPI [10], enhancing the wettability and decreasing the swelling ratio of protein-based films. These biopolymer networks can accelerate the diffusion of water molecules, resulting in a decrease in swelling behavior and water solubility of the film. Considering previous studies, the improvement in polysaccharide or protein-based film properties is principally based on the film composition and molecular structure, resulting in the formation of both covalent and hydrogen bonding in the hydroxyl, amine, and amino groups of polysaccharides and protein polymers.

Hence, an alternative source of protein derived from honey bee broods (larvae and pupae) is a new film component serving as a functional additive for increasing the polysaccharide-based film properties, particularly the water resistance properties. Honey bee broods (larvae and pupae) are nontoxic and very rich in nutrients, with high protein and fat contents [15]. Additionally, the proximate composition of larval and pupal homogenates was 45.9% protein, and the essential amino acids of larvae and pupae were leucine (6.6 g/kg), lysine (5.8 g/kg), and isoleucine (4.3 g/kg), respectively [16,17]. However, there are no reports on the utilization of honey bee brood protein as an additive for polysaccharide biopolymer gel films, which needs to be investigated. Therefore, this research aimed to develop and characterize CMS/CS/PT biopolymer films crosslinked with DCMC and reinforced with BBP. The primary objectives are as follows: (i) to investigate the influence of BBP concentration on the mechanical, thermal, and water vapor barrier properties of the films and (ii) to understand how BBP interacts with DCMC to improve film properties. The findings from this study provide valuable insights into the development of high-performance, biodegradable biopolymer films suitable for packaging applications.

## 2. Results and Discussion

### 2.1. Amino Acid Composition of BBP

The amino acid composition of the BBP is shown in Table 1. The extraction yield and purity of BBP are 9.29% and 73.42%, respectively. Compared with the basic nutritional requirements specified by FAO standards, BBP has insufficient amounts of essential amino acids [18]. The amino acids present are threonine, valine, isoleucine, leucine, methionine, lysine, and histidine. The percentage of essential amino acids (41.78%) was less than the percentage of nonessential amino acids (58.22%). Therefore, it is not appropriate for human consumption. The total hydrophobic amino acid concentration of phenylalanine, valine, leucine, isoleucine, alanine, methionine, and proline was 60.99%, exceeding the hydrophilic amino acid concentration in BBP. Proline had the highest concentration of amino acids, approximately 1.51 mg/g on a dry basis. Similarly, the concentration of essential amino acids in honey bee (*A. mellifera*) larvae and pupae was shown to be lower than that of nonessential amino acids [19]. The high concentration of hydrophobic amino acids in honey bee brood protein (BBP) enhances the mechanical strength and moisture resistance of biopolymer films by promoting stronger intermolecular interactions and reducing water absorption. These hydrophobic amino acids, such as leucine and valine, contribute to a denser and more cohesive film structure, improving its barrier properties against moisture transmission [20,21]. Honey bee brood protein (BBP) exhibits an incomplete essential amino acid profile, as it lacks six key amino acids: tryptophan, cysteine, glutamine, asparagine, tyrosine, and arginine, limiting its suitability for human nutrition. The composition of BBP is highly dynamic, influenced by multiple factors including developmental stage, where amino acid requirements shift between larval and pupal phases, genetic differences among bee subspecies, and environmental conditions such as the availability of pollen and nectar sources [22,23]. The amino acid composition of honey bee brood protein (BBP) is well-balanced and comparable to high-quality protein sources, with notably high levels of hydrophobic amino acids such as leucine and valine [15,24]. This composition improves the structural integrity and water resistance of biopolymer-based films, enhancing their mechanical properties and flexibility. Compared to plant-based proteins, BBP demonstrates superior film-forming capabilities, making it a promising candidate for the development of sustainable food packaging materials [20,21].

### 2.2. Carboxymethyl Starch Characterization

Carboxymethyl starch (CMS) is synthesized through alkalization and etherification reactions or carboxymethylation [25]. The carboxymethylation process involves the substitution of a hydroxyl group (-OH) in natural starch with a carboxymethyl group (-CH2COO-) to create Na-carboxymethyl starch or carboxymethyl starch (CMS) [26]. In the experiment, rice starch was used to synthesize and to characterize its properties before producing the tertiary biopolymer blend film, resulting in high purity and a high degree of substitution (DS). The CMS had a purity of 93.83 ± 0.12%, and the byproduct contents of sodium chloride and sodium glycolate were 1.56 ± 0.03% and 4.61 ± 0.38%, respectively. Similarly, the work accomplished by Jainan et al. [27] illustrated that the carboxymethylation reaction of rice starch could conduct the high DS and low sodium chloride content, which were 98.60 ± 0.55% and 1.54 ± 0.02, respectively. However, the CMS synthesis process can be affected by the over demand of sodium hydroxide (NaOH) and monochloroacetic acid (MCA), introducing into the reaction mixture with starch, which makes it have high DS and high impurity. Hence, when an excessive amount of monochloroacetic acid is introduced, it will undergo a reaction with NaOH, leading to the formation of sodium glycolic acid [26].

### 2.3. Composite Film Characterization

#### 2.3.1. Color and Optical Properties

**Figure 1:**
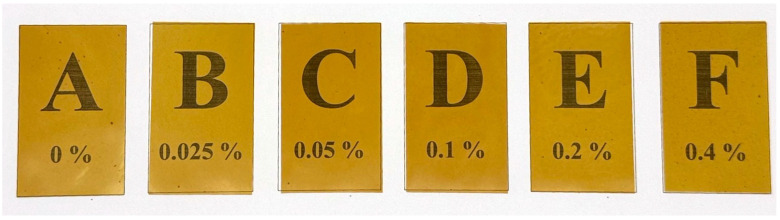
The appearance of CMS/CS/PT gel films with different concentrations of honey bee brood protein: 0% (**A**), 0.025% (**B**), 0.05% (**C**), 0.1% (**D**), 0.2% (**E**), and 0.4% (**F**) (*w*/*v*).

The optical properties of packaging films are crucial factors that directly impact the consumer’s perception of the product, as consumers may visually inspect both the appearance of the product in its packaging and the features that increase its shelf life [28,29]. The color difference was determined from the values of L*, a*, b*, and ∆E. The color and opacity of the different BBP concentrations of the polysaccharide gel films are shown in Table 2, and the appearances of polysaccharide gel films are shown in Figure 1. The L* of CMS/CH/PT with different concentrations of BBP was significantly different from that of the control film, which was in the range of 77.19–80.05. A higher L* was found with the addition of the BBP film. As a result, adding BBP improved the brightness of the polysaccharide gel film. The b* value, which represents the blue (negative)–yellow (positive) color, decreased significantly as the BBP concentration in the polysaccharide gel films increased. Moreover, the a* value, which represents green (negative) and red (positive) color, also slightly decreased. The addition of honey bee brood protein (BBP) affects the color attributes (L*, a*, b*) of polysaccharide gel films by increasing brightness (L*) while decreasing yellowness (b*) and redness (a*). The total color change (∆E) of the CMS/CS/PT film ranged from 29.36–35.19. The transparency of a film is an important property for food packaging, as it enables the visibility of the packaged product. This change enhances the film’s visual appeal and light barrier properties, which can influence consumer perception of freshness and quality in food packaging [30,31]. Optimizing BBP concentrations can balance these optical properties to improve both functionality and marketability [32,33]. An established indicator of film transparency is opacity, where a higher opacity corresponds to a lower transparency [16]. The opacities of the CMS/CH/PT films ranged from 3.24–4.22 mm^−1^. The highest opacity was observed for the CMS/CS/PT film with 0.4% (*w*/*v*) BBP, which was greater than that of the control film. No strong difference was observed between the opacities of the polysaccharide gel films incorporated with BBP, but only a tendency toward increasing opacity with increasing amounts of BBP was observed. Increasing BBP concentration in polysaccharide gel films enhances opacity, improving light barrier properties and reducing oxidative degradation, which may extend the shelf life of packaged foods [30,31]. However, excessive opacity can affect consumer perception, as transparency is often associated with product visibility and freshness [32,33]. Maintaining an optimal balance between opacity and transparency is crucial to ensuring both functional protection and market appeal. Natural additives and biopolymer modifications can be employed to enhance protective properties while preserving consumer acceptance [20,21].

#### 2.3.2. Mechanical Properties

The mechanical properties of biopolymer films are affected by how the polymer chains interact with each other and with other molecules. The crucial characteristics to consider are the tensile strength (TS) and elongation at break (EB). Tensile strength (TS) and elongation at break (EB) are crucial indicators of the strength and ability of a film to stretch before breaking [34]. The improvement in the mechanical characteristics of the polysaccharide gel films indicates an increase in their ability to endure stress during storage and packing, hence preventing the possibility of penetration and tearing in the composites. Table 3 displays the mechanical properties of the polysaccharide gel films at varying concentrations of BBP. The addition of BBP (0.025% and 0.05% *w*/*v*) resulted in decreased tensile strength and elongation at break and continued to drop when BBC was above 0.1% *w*/*v* afterward. This phenomenon is typical for biopolymer films, in which the addition of immiscible substances causes structural discontinuities in the polymer network and a decline in the overall cohesive forces of the matrix [35], confirmed by the SEM image of the CMS/CS/PT film at different BBP concentrations.

The highest tensile strength of the CMS/CS/PT film is 0.1% *w*/*v* BBP (7.73 ± 0.09 MPa), which is not significantly different from that of the sample without BBP (7.95 ± 0.19 MPa), accompanied by an increase in elongation at break. The maximum value of elongation at break was found for 0.1% *w*/*v* BBP of CMS/CS/PT, which reached 32.23%. However, it was lower than that with no added BBP film (34.90%). For this reason, it might be that the hydroxyl group of BBP is adequate for forming hydrogen bonds with CMS/CS/PT, resulting in stronger interfacial adhesion and a more complex crosslinked biopolymer structure between BBP and the CMS/CS/PT film. Moreover, the occurrence of crosslinking between BBP, DCMC, and polysaccharide biopolymer (CMS/CS/PT) can result in reduced elongation at break of the biopolymer gel film, causing a denser and more rigid gel film structure [36]. Similar to the present study, the addition of DCMC at any DCMC level causes an increase in tensile strength and a decrease, suggesting the occurrence of crosslinking between gelatin and DCMC [37]. The data observed that the lowest tensile strength and elongation at break were found at the maximum concentration of BBP (0.4% *w*/*v*) because it exhibits greater deflection and more inhomogeneous surface from BBP immiscible particles in blended gel film (Figure 1), making it more susceptible to breaking.

To characterize the structural stability of polysaccharide gel film during swelling in water, the result was evaluated by TS and EAB of CMS/CS/PT film after soaking in water for 24 h and presented the decline of both mechanical properties (Table 3), resulting in the highest TS and EAB of CMS/CS/PT film at 0.1% (*w*/*v*) of BPP. They were 0.35 MPa and 15.45%, respectively. The contact between CMS/CS/PT and BPP can be destroyed by the distribution of more water molecules in the biopolymer network’s free volume. Notably, the many hydrophilic groups in DCMC that can adsorb moisture lead to plasticization of water molecules to the film, canceling part of the crosslinking effect by DCMC. Moreover, it causes the mobility of the polysaccharide and protein chains, which decreases the TS and EAB of polysaccharide gel film [38,39].

#### 2.3.3. Water Susceptibility

The water sensitivity of a biopolymer film is a crucial characteristic, as it might impact its application and utilization. The moisture content, water absorption, thickness, swelling, and water solubility of the polysaccharide gel films are shown in Table 4. The moisture contents of the different concentrations of BBP-based polysaccharide gel films did not significantly differ and were in the range of 6.92–7.75%. However, the BBP-containing polysaccharide gel films presented a lower moisture content than the non-BBP-containing polysaccharide gel films. Because proline is nonpolar due to it containing a ring structure that includes the amine group and exists in zwitterionic form, it makes it quite soluble in water as well as good for hydrophobicity [15,40].

The water contact angle corresponds to the intermolecular interactions and chemical crosslinking in a polymeric film [41,42]. Typically, a surface with a contact angle less than 90° is referred to as hydrophilic [43]. The results revealed that the contact angle of the control film was measured to be 76.18°. Note that, at a concentration of 0.1% *w*/*v*, the contact angle of CMS/CS/PT with BBP reached its greatest value of 83.68°, suggesting that the addition of BBP may enhance the hydrophobic properties of the film surface and form a complex structure by conjugating with a DCMC crosslinker. Moreover, the aldehyde group at one end of DCMC can form strong interactions with the amine functional group of the adjacent lysine and proline residues in the BBP, where it protects the polar groups from H_2_O molecules to some extent [44].

**Figure 2:**
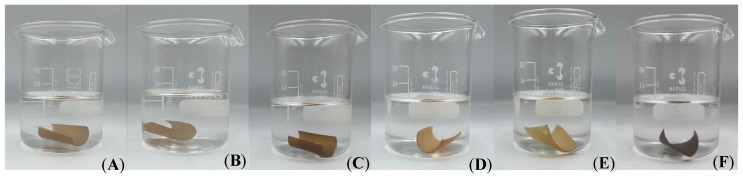
Structural stability of CMS/CS/PT gel films with varying concentrations of honey bee brood protein: 0% (**A**), 0.025% (**B**), 0.05% (**C**), 0.1% (**D**), 0.2% (**E**), and 0.4% (**F**) *w*/*v*, after soaking in water for 24 h.

In fact, in the honey bee brood nonessential amino acids profile, there are around 58.22% that might lead to the higher hydrophobicity of this CMS/CS/PT film. Similar to the contact angle, the CMS/CS/PT with BBP film had the lowest water solubility of all the polysaccharide gel films at a concentration of 0.1% *w*/*v*, at 29.16%. Additionally, throughout the solubility test, all the polysaccharide gel film samples maintained their initial form and good structural stability without any gel film deterioration (Figure 2). As a DCMC crosslinker, DCMC addition increases the cohesion of the polymer matrix and increases the crosslinked structure of the polymer by forming hydrogen bonds with hydrophilic groups in BPP and polysaccharide-based gel film structures. This reduces the number of film interactions with water. For example, the feather keratin/DCMC film’s solubility dropped to 45% at 30% wt (glycerol) and 5% wt (DCMC) as the DCMC concentration increased, which was less than that of the control film [43]. As with the film, solubility declined as DCMC content rose, indicating that sericin’s stability was successfully increased by DCMC crosslinking [14].

The water swelling behavior of the polysaccharide gel film was evaluated in terms of the water absorption capacity and thickness swelling (Table 4). Figure 2 shows the structural stability and thickness swelling of CMS/CS/PT film at different BBP concentrations. Typically, the water absorption capacity depends on the presence of hydrophilic groups, the free volume, and the crosslinking density [45]. The water absorption and thickness swelling of CMS/CS/PT film increased significantly with increasing BBP content until 0.1% *w*/*v* and at this concentration illustrated that a gel formation was observed at low concentrations of crosslinker. The increased amount of BBP, which can cause a drop in free volume but increase the crosslinking density, caused a decrease in water absorption and thickness swelling once the BBP concentration was above 0.1% *w*/*v*. By bonding with the amorphous structure of CMS and PT, a Schiff base-linked product from a nucleophilic addition condensation reaction between the amine group of the chitosan and the aldehyde group of the DCMC and a polyelectrolyte complex ionic interaction between CS and PT creates a 3D gel network in the gel film, increasing its free volume and capacity to hold large volumes of water without film deformation (Figure 2) [14,46].

The gel film expands in an aqueous medium because of the absorption of water, which is facilitated by the complex van der Waals interactions between water and the numerous hydrophilic functional groups in the gel film component and crosslinker backbone [38]. The diffusion of water into the polymeric film network results in swelling of the gel film, which is counterbalanced by elastic retractive forces. Additionally, the film’s expansion behavior can be influenced by the degree of crosslinking among the various polymer chains, which is how DCMC interacts with polysaccharides and BBP.

To determine the water vapor barrier of CMS/CS/PT/BBP film, water vapor permeability (WVP) is a critical parameter when determining the preservation capabilities of food packaging films. In general, therefore, the WVP is importantly affected by the hydrophilic properties of polymer composition and the internal tortuosity in the microstructure of the polymer matrix [47]. The WVP resulting from the incorporation of BBP CMS/CS/PT films was lower than that resulting from the omission of the film, as illustrated in Table 4. The WVP significantly continued to decrease as the concentration of BBP increased within the range of 4.3–3.11 × 10^−6^ g/m·day·Pa, whereas the WVP of the control film was 4.50 × 10^−6^ g/m·day·Pa. The result of CMS/CS/PT/BBP film has mainly two factors for improving film barrier properties, which are the following: the DCMC crosslinking effect and the rising of hydrophobic parts. The network structure created by the DCMC-induced crosslinking action increases the density of the film microstructure and prevents the diffusion of water molecules within the film [39]. According to a previous study, the WVP values of gelatin films decreased as DCMC increased. This suggests that DCMC can lessen the water sensitivity of gelatin-based films, and it may be because compact protein networks in gelatin–DCMC films provide a more tortuous path to the water molecules [37,48]. In the case of BBP blending, which contains a high proportion of nonpolar amino acids, this issue was effectively resolved by introducing hydrophobic amino acids onto the film surface, thereby reducing the hydrophilicity of polymer films [49]. For this reason, at higher concentrations of BBP, polysaccharide–DCMC–protein interactions increased, resulting in lower mechanical properties but improving WVP due to greater amounts of hydrophobic BBP. In general, packaging film materials with a lower WVP provides superior protection against the infiltration of moisture from the surrounding environment and water loss in packaged food. The WVP resulting from the incorporation of BBP CMS/CS/PT films was lower than that resulting from the omission of the film, as illustrated in Table 4. The WVP significantly continued to decrease as the concentration of BBP increased within the range of 4.3–3.11 × 10^−6^ g/m·day·Pa, whereas the WVP of the control film was 4.50 × 10^−6^ g/m·day·Pa. BBP, which contains a high proportion of nonpolar amino acids, was able to effectively resolve this issue by introducing hydrophobic amino acids onto the film surface, thereby reducing the hydrophilicity of polysaccharide-based films. In addition, at higher concentrations of BBP, polysaccharide–DCMC–protein interactions increased, resulting in lower tensile strengths but improving WVP due to greater amounts of hydrophobic BBP.

#### 2.3.4. FTIR Analysis

**Figure 3:**
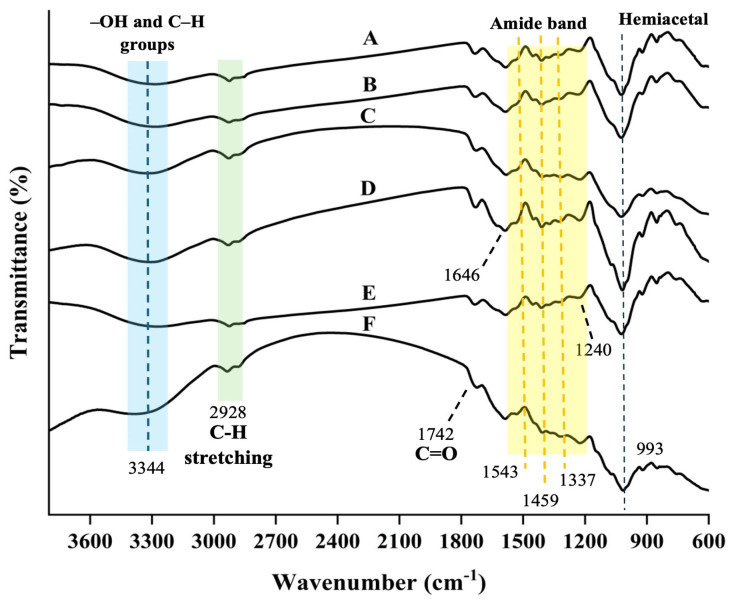
FTIR spectra of CMS/CS/PT composite film with different concentrations of honey bee brood protein: 0% (**A**), 0.025% (**B**), 0.05% (**C**), 0.1% (**D**), 0.2% (**E**), and 0.4% (**F**) *w*/*v*.

ATR-FTIR spectra were used to analyze the changes in the functional groups of biopolymer gel film (CMS/CS/PT film) at different concentrations of honey bee brood protein with DCMC used as a crosslinking agent, as shown in Figure 3. The results revealed peaks at 3446 cm^−1^ and 2928 cm^−1^, which suggested the presence of –OH and C–H groups in the CMS and chitosan. Moreover, the addition of BBP is a protein base component in biopolymer gel film, resulting in the N–H stretching vibration (amide A) of proteins, which typically absorbs in the spectral range from 3500 to 3300 cm^−1^ [50], and the vibration around 2927 cm^−1^ corresponds to C–H stretching of methyl and methylene groups of both proteins and lipids [51].

In the CMS, the asymmetric stretching vibration and symmetric stretching vibration of –COO- were indicated by the intense bands at 1596 cm^−1^ and 1419 cm^−1^, respectively [52,53]. The C=O, N–H, and C–N bonds of the amide band of the core structure of chitosan and BBP, respectively, are represented by the peaks that are seen at 1646, 1543, and 1240 cm^−1^ (Figure 3) [54,55]. Similarly, C=O and C–N stretching vibrations (amide I) are responsible for the medium intensity vibration at 1636 cm^−1^. These vibrations may be linked to peptides or globular proteins found in honey bee protein. Furthermore, the N–H deformation and C–N stretching vibrations (amide II) of peptides and other protein-based components are referred to by the spectrum region between 1510 and 1544 cm^−1^ [51].

Considering the spectral region between 1470 and 1240 cm^−1^, it is characterized by a series of weak signals at 1459 due to CH_2_ bending, and at 1337, that is the C–H deformation vibration, which overlaps with C–N stretchings (amide III). These absorption bands are attributed to the free amino acids of protein [56]. In work accomplished by Wellner et al. (1996), it was reported that these signals are highly specific for proline, given that proline represents a predominant free amino acid in the honey bee protein [57]. In this case, the band at 1742 cm^−1^, associated with C=O in the ester bonds of pectin and the appearance of the aldehyde group, suggested that DCMC had been crosslinked [58,59,60]. The FTIR spectra of the polysaccharide gel film verified the absence of any new covalent bonds formed between chitosan, CMS, and pectin, as the FTIR spectra of the samples were nearly identical.

#### 2.3.5. Thermal Properties

**Figure 4:**
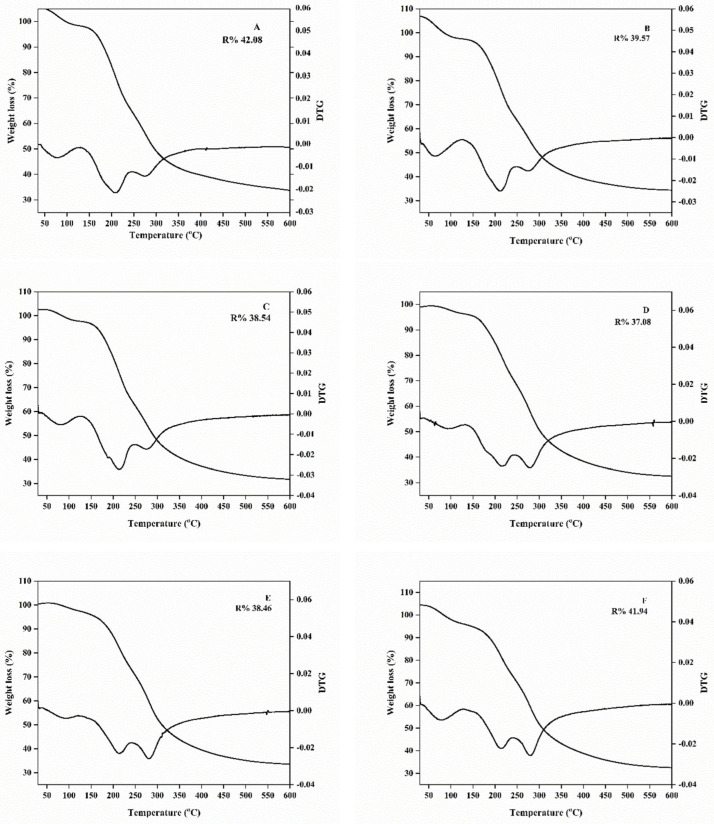
TGA thermograms of CMS/CS/PT composite film with different concentrations of bee brood protein: 0% (**A**), 0.025% (**B**), 0.05% (**C**), 0.1% (**D**), 0.2% (**E**), and 0.4% (**F**) *w*/*v*.

**Figure 5:**
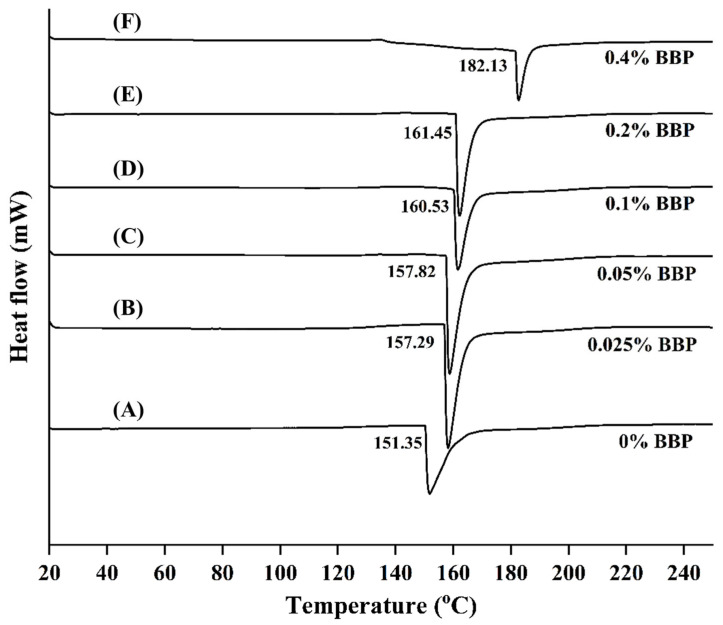
DSC thermograms of CMS/CS/PT composite film with different concentrations of honey bee brood protein: 0% (**A**), 0.025% (**B**), 0.05% (**C**), 0.1% (**D**), 0.2% (**E**), and 0.4% (**F**) *w*/*v*.

The thermal properties of the CMS/CS/PT and CMS/CS/PT with BBP films are shown in Figure 4 and Figure 5. The TG and DTG curves demonstrate that all the polysaccharide gel films exhibited similar patterns of weight reduction, revealing the occurrence of three stages of thermal degradation, as shown in Figure 4. There are three main weight loss steps. The initial degradation, occurring between 50 °C and 150 °C, can be attributed to the evaporation of absorbed water and loosely bound and structurally bound water molecules, including volatile organic compounds [59,60,61,62,63], as indicated by the moisture content (%, MC), which is approximately 8%, as presented in Table 4. The significant decrease in weight observed between temperatures of 150 and 350 °C may be due to the thermal disintegration of intermolecular hydrogen bonds, as well as the breakdown of oxygen-containing groups such as hydroxyl and carboxyl groups and other side chains [64]. The second stage of mass loss occurred within the temperature range of 170–245 °C and achieved the highest rate of decomposition at approximately 210.79–216.76 °C. Finally, the third stage occurred at a temperature range of 250–320 °C and the maximum decomposition rate was 277.50–284.39 °C, which corresponds to polysaccharide gel film decomposition temperatures, and they tended to rise with an increase in BBP concentration, but they were not significantly different at (0.1–0.4%; *w*/*v*). This implies that the addition of BBP at optimal concentration can improve the thermal stability of polysaccharide gel film. The result is implied that the addition of BBP at optimal concentration can improve the thermal stability of polysaccharide gel film due to BBP’s high proline content and its degradation temperature around 298.47 °C [40].

Furthermore, the char residue of the CMS/CS/PT film was approximately 34.65%, and that of the CMS/CS/PT film with BPP was in the range of 32.88–35.06%. The char residue of the film is not significantly different. A comparison between CMS/CS/PT film with BPP and other polysaccharide or protein-based fibers conjugated with DCMC reveals similar thermal degradable behavior, as determined by TGA analysis. For instance, all gelatine-DCMC films exhibited three main stages of weight loss, and their degradation temperature tended to rise with an increase in DCMC, resulting in approximately 315 °C [37]. This is attributed to the DCMC crosslinking effect, which leads the biopolymer film to be denser and stiffer, and in turn, the thermal stability is improved. Similar to the addition of DCMC as a functional crosslinking in gelatin–DCMC–montmorillonite (MMT) films, it played an important role in contributing to the complex gelatin structure and motivated the good dispersion of MMT in the gelatin matrix, creating high thermal stability of the gelatin film [65].

The DSC thermograms of the polysaccharide gel films are shown in Figure 4. The melting temperature (Tm) of the control film (CMS/CS/PT) without BBP is 151.35 °C. The polysaccharide gel film containing 4% *w*/*v* of BBP presented the greatest melting temperature (182.13 °C). Moreover, the difference in BBC concentration was added in polysaccharide gel film that had strongly affected the Tm of the CMS/CS/PT film, increasing from 157.29 to 182.13 °C. The Tm trend was positively correlated with increasing amounts of BBP because proline was the only proteinogenic secondary amino acid that had a five-membered ring, making it form large side groups in the polymer chain. In addition to proline’s ring structure, which can enhance the thermal properties of CMS/CS/PT/BPP film, the inclusion of DCMC as a crosslinker accelerates the increase in thermal stability by forming a strong polymer network structure in the CMS/CS/PT with the BBP matrix. This is confirmed by the high addition of DCMC in gelatin film, improving the film’s thermal stability [37]. Basically, crosslinking is useful for biopolymer film produced from polysaccharide and protein blended. It also improves the heat and dimensional stability [66]. Similar results: the addition of crosslinkers can increase the covalent bonding to establish stronger intermolecular covalent bonds and to attain closer molecular packing and reduced polymer mobility or rotation, illustrating high glass transition, high melting temperature, and high degradation temperature [67].

#### 2.3.6. Scanning Electron Microscopy (SEM)

**Figure 6:**
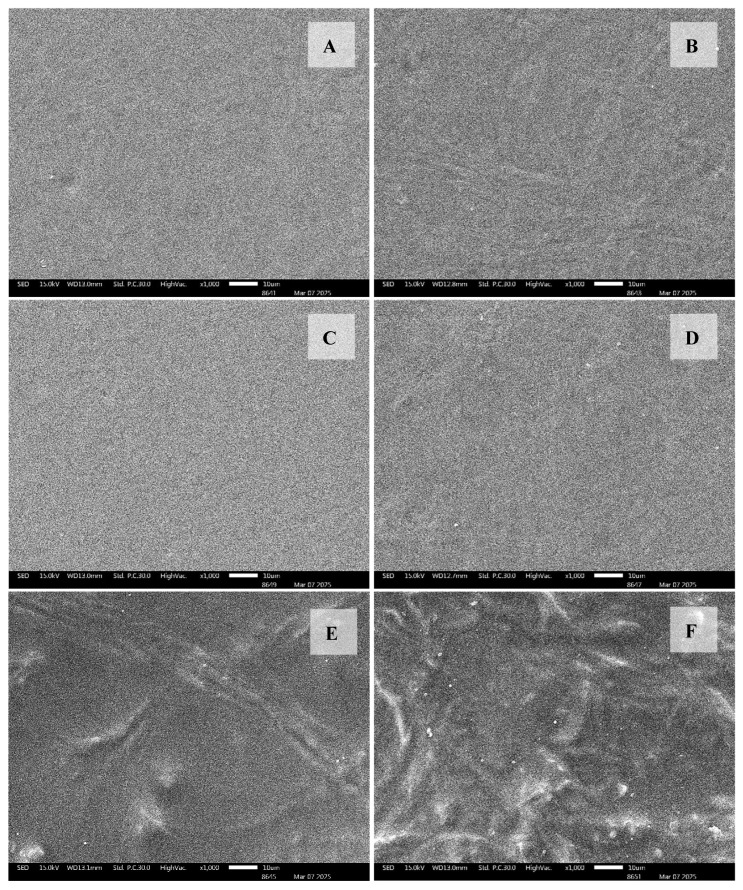
Scanning electron microscope (SEM) of CMS/CS/PT gel films with different concentrations of honey bee brood protein (BBP): 0% (**A**), 0.025% (**B**), 0.05% (**C**), 0.1% (**D**), 0.2% (**E**), and 0.4% (**F**) *w*/*v*.

The SEM picture (Figure 6) of the surface of biopolymer gel film blending shows the effect of BBC addition on the surfaces of CMS/CS/PT gel films. Comparing with the control gel film, the CMS/CS/PT gel film exhibited a smooth structure, a homogeneous and dense microstructure at minimal BBP concentration (0.025–0.01% *w*/*v* (B–D)), except for the presence of an unsmooth surface at 0.2–0.4% (*w*/*v*) (E–F), as well as a few DCMC particles arising from the crystallization of salts during film drying [46]. To determine the uniformity and biopolymer phase separation of CMS/CS/PT gel films, it clearly occurs at the highest BBP concentration (0.4% *w*/*v* (F)) due to it developing irregular structures and protein aggregation with increasing BBP content. This phenomenon indicated that the amino acid in BBC has side chains that do not like to reside in water, causing the low compatibility between the biopolymer gel film components [68].

## 3. Conclusions

Honey bee brood protein (BBP) was employed as an additive in polysaccharide-based films (CMS/CS/PT) to affect their gel film character. The incorporation of BBP significantly influenced the hydrophilicity, water susceptibility, mechanical strength, and thermal stability of films, with optimal improvements observed at a concentration of 0.1% (*w*/*v*) BBP. The water contact angle increased from 76.18° (control) to 83.68° at 0.1% (*w*/*v*) BBP, indicating enhanced hydrophobicity. Additionally, water vapor permeability (WVP) decreased from 4.50 × 10^−6^ g m^−1^ d^−1^ Pa^−1^ (control) to 3.93 × 10^−6^ g m^−1^ d^−1^ Pa^−1^ at 0.1% BBP, demonstrating improved film barrier properties. Water absorption increased at lower BBP concentrations, peaking at 369.01% before declining at higher BBP levels due to increased crosslinking density. Thickness swelling followed a similar trend, reaching 248.75% at 0.1% BBP. In this case, the mechanical properties showed that the tensile strength of the films reached a maximum value of 7.73 MPa, while maintaining good elongation at break (32.23%). In contrast, increasing BBP concentration beyond 0.1% (*w*/*v*) led to an increase in thermal properties, especially the melting temperature (T_m_), which rose from 151.35 °C (control) to 182.13 °C at 0.4% (*w*/*v*) BBP, and the degradation temperature was 283.11 °C, indicating enhanced thermal resistance. Fourier transform infrared spectroscopy (FTIR) verified the formation of Schiff base linkages and hydrogen bonding between BBP and the polysaccharide matrix, contributing to improved film integrity. SEM images showed that BBP concentrations up to 0.1% (*w*/*v*) produced smooth, homogeneous film surfaces, while higher concentrations resulted in phase separation and aggregation. Therefore, BBP has the ability to function as a film additive, which has the potential to increase the quality of the polysaccharide gel film used in food packaging or active food packaging, such as humidity control packaging.

## 4. Materials and Methods

### 4.1. Materials

The raw material used in the present research consisted of drone broods (larva to prepupal stages) of the honey bee species *Apis mellifera* L. collected from an apiary located in Phare, Thailand. Commercial chitosan powder (CS) with a 92.50% degree of deacetylation was obtained from the king crab shell (Union Science Co., Ltd., Chiang Mai, Thailand). High-methoxyl pectin with a degree of esterification 75% was purchased from Union Science Co., Ltd. (Chiang Mai, Thailand). Carboxymethyl starch (CMS) with an amylose content of 28.42% was prepared from a rice starch (Bangkok Inter Food Co., Ltd., Nakornpathom, Thailand). Dialdehyde carboxymethyl cellulose (DCMC) with an aldehyde content of 70.99% was prepared on the basis of previous research by Tachai and colleagues [69]. All chemical reagents were analytical grade, such as ethanol (RCI Labscan, Bangkok, Thailand), hexane (RCI Labscan, Bangkok, Thailand), monochloro acetic acid (Loba Chemie, Mumbai, India), and propanol (RCI Labscan, Bangkok, Thailand).

### 4.2. Carboxymethyl Starch (CMS) Synthesis

Approximately 10 g of rice starch was weighed and poured into sodium hydroxide solution (30% *w*/*v*) for the alkalization reaction. After that, isopropanol was added to the mixture, which was stirred at 500 rpm at ambient temperature. Next, sodium monochloroacetic acid was added to the rice starch mixture (1.6 M), and the mixture was incubated at 55 °C for 4 h. At the end of the reaction, the starch slurry was filtered, suspended in ethanol, and neutralized with acetic acid. Following filtration, the slurry was dispersed again in 80% ethanol until the silver nitrate test for chloride of the filtrate was negative. The carboxymethyl starch was dried in an oven at 40 °C for 8 h until drying was complete at 5% moisture content. The degree of substitution (DS), purity of the CMS, NaCl content, and degradation temperature were characterized [27].

### 4.3. Honey Bee Brood Protein Extraction (BBP)

Briefly, the raw bee brood was blended with water and steamed for 1 min. After that, the pH of the filtrate of the bee brood mixture was adjusted to 8, and the mixture was incubated on a shaker for an hour at room temperature. After centrifugation (10 min, 7500 rpm, 4 °C), all protein aggregates were collected, and fat was removed with hexane. The crude protein of each bee brood was subsequently filtered and dried overnight at room temperature. The amino acids of BBP were analyzed.

### 4.4. Polysaccharide Gel Film Preparation

The carboxymethyl starch (CMS), chitosan (CS), and pectin (PT) composite films were prepared via solution casting. Three biopolymers were individually dissolved to prepare a ternary composite film. The CMS and PT solutions were prepared by dissolving 2% (*w*/*v*) in distilled water at 80 °C for 45 min with continuous agitation. Next, a chitosan solution with a concentration of 2% (*w*/*v*) was prepared by dissolving it in 0.1 M lactic acid. The three solutions were mixed in equal proportions, and 0.5% (*v*/*v*) glycerol was used as the plasticizer. DCMC, which was used as a crosslinking agent, was added at a concentration of 0.1% *w*/*v* to the CMS/CS/PT film solution. After that, crude bee brood protein (BBP) was added at different concentrations (0–0.4%, *w*/*v*) to the CMS/CS/PT film. The CMS/CS/PT film solutions were cast onto Teflon glass plates and dried at 45 °C overnight. The CMS/CS/PT films were subjected to a controlled relative humidity chamber at a temperature of 25 ± 1 °C and a relative humidity of 52% for a minimum of 24 h prior to their characterization.

### 4.5. Polysaccharide Gel Film Physical Properties

#### 4.5.1. Film Thickness

The film thickness was determined by directly measuring the film at five random points on each film via a micrometer (Miyamoto, Tokyo, Japan) with an accuracy of 0.001 mm.

#### 4.5.2. Surface Color Measurements

The color of each film was determined via the CIE Lab values, which are L* (lightness), a* (red/green), b* (blue/yellow), and ΔE (total color difference), via a colorimeter (3NH Technology Co., Ltd., Model NR 145, Guangdong, China) with a white color plate (L* = 91.93, a* = 0.43, and b* = −1.24) as a standard background for the color measurement. The total color difference between the samples was determined using Equation (1):

(1) $$∆E=\sqrt{∆L^{2}+∆a^{2}+∆b^{2}}$$

where ΔL, Δa, and Δb represent the changes in lightness, redness, and yellowness of the film sample compared to the standard white plate.

The Yellow Index (YI) is a numerical metric used to quantify the degree of yellowness in a material. It is particularly relevant in industries dealing with polymers, textiles, coatings, and paper. The YI value indicates how much a material’s color deviates from pure white or colorless, trending toward a yellow appearance (ASTM E313-20) [70]. It can be calculated using the following formula:

(2) $$YI=\frac{142.86\times b}{L}$$

where L represents lightness value of the film, b represents yellowness value of the film, and 142.86 is standard scaling factor.

#### 4.5.3. Opacity

An ultraviolet-visible (UV-vis) spectrophotometer (V-730, Jasco, Tokyo, Japan) was used to measure the transmittance of the film segments, which were cut into lengths of 1 × 4 cm^2^. The absorbance values at 600 nm were recorded, and the opacity was determined via Equation (3):

(3) $$Opacity=\frac{{Abs}_{600}}{X}$$

where Abs_600_ is the value of absorbance at 600 nm and X is the film thickness (mm).

#### 4.5.4. Moisture Content of the Film

The composite films were cut into 2 × 2 cm pieces, and the initial weight of the film was recorded. Afterward, the film samples were dried in a hot air oven at 105 ± 1 °C for 24 h. The moisture content was determined by calculating the quantity of water that was removed from the film, expressed as a percentage. The experiment was carried out five times, and the values were determined as the average for each sample film.

#### 4.5.5. Water Absorption and Thickness Swelling

The water absorption and thickness swelling of the films were evaluated by soaking them in water at 25 °C. The films were conditioned before testing by keeping them at a constant relative humidity (RH) of 0% and a temperature of 25 °C. The films were then removed and weighed at 2 and 24 h to measure the weight gain of the film, including the thickness of the film. The moisture absorption and thickness swelling were determined via Equations (2) and (3):

(4) $$\mathrm{Water}\;\mathrm{absorption}\;(\%)=\frac{\left(W_{f}-W_{i}\right)}{W_{i}}\times 100,$$

where W_i_ is the initial weight of the film and W_f_ is the weight of the film at 24 h.

(5) $$\mathrm{Thickness}\;\mathrm{swelling}\;(\%)=\frac{\left(t_{f}-t_{i}\right)}{t_{i}}\times 100,$$

where t_i_ is the initial thickness of the film (mm) and t_f_ is the thickness of the film at 24 h (mm).

#### 4.5.6. Water Contact Angle

The contact angle of water on the film surface was determined at a temperature of 25 °C via a goniometer equipped with a drop shape analysis system (DSA) developed by KRÜSS DSA30 in Hamburg, Germany. The contact angle was measured at the 120 s time point after the water droplet made contact with the film surface. The data were tested in five replications and were thereafter recorded.

### 4.6. Water Vapor Permeability (WVP)

The ASTM E96-00 [71] was employed to measure water vapor permeability (WVP) gravimetrically at 25 °C. The composite films were cut into circles with a diameter of 7.40 cm and a test area of 33 cm^2^. The film samples were then applied to each permeation cell after 10 g of anhydrous silica gel (0% RH) was added. Each permeation cell was subsequently placed in a desiccator that contained a saturated Mg(NO_3_)_2_ solution at the bottom to maintain a constant relative humidity (RH) of 52% at 25 °C. Every day, the weight of the permeation cell was recorded as a function of time. Subsequently, linear regression was implemented to determine slopes (weight change vs. time). Finally, the following equation was employed to determine the WVP (g/m day Pa):

(6) $$WVTR=\frac{G}{t\times A}$$

where G represents the change in weight (g), t is the time (days), and A is the test area (m^2^).

(7) $$WVP=\frac{WVTR\times L}{∆P}$$

where the water vapor transmission rate (WVTR) was defined as the slope (g/day) divided by the transfer area (m^2^). At a test temperature of 25 °C, ΔP represents the water vapor pressure difference across the film (Pa), and L represents the film thickness (m). Triplicate measurements were carried out.

### 4.7. Polysaccharide Gel Film Mechanical Properties

A universal test machine up to 1 kN (LS 1, Lloyd, West Sussex, UK) was used to evaluate the mechanical properties of the films in terms of tensile strength and elongation at break, following the standard method ASTM D 882-83 [72]. The film was a strip 10 mm in width and 70 mm in length. The thickness of the films was measured via a digital micrometer (Mitutoyo, Tokyo, Japan) at five random points. The initial grip separation was set at 50 mm, and the samples were pulled apart at a crosshead speed of 0.5 mm/s. Ten replicates of each film were tested. The tensile strength (TS) was determined by dividing the maximum force (N) that the film sample can withstand before breaking by its cross-sectional area. The elongation at break (%) was determined by dividing the distance at break by the initial length of the sample and then multiplying the result by 100%.

### 4.8. Fourier Transform Infrared (FT-IR) Spectroscopy

The FTIR spectra of different films were acquired via an attenuated total reflectance (ATR) system. FTIR spectral analysis was carried out on a Jasco FTIR instrument (4700, Tokyo, Japan). The spectra were recorded in absorbance mode from 3800 to 600 cm^−1^ with a resolution of 4 cm^−1^. Three replicates were collected for each sample [73].

### 4.9. Polysaccharide Gel Film Thermal Properties

The melting temperature (T_m_) was obtained via differential scanning calorimetry (DSC) (Mettler-Toledo823, Mettler-Toledo Inc., Schwerzenbach, Switzerland). The average weight of the film ranged from 5–10 mg, and the film was placed in an aluminum pan. The samples were investigated by heating and cooling cycles from 30 to 250 °C with a heating rate of 10 °C/min. The experiments will be conducted at least three times for each material composition.

The thermal decomposition of the composite film was examined via TGA. The samples were weighed to 8–10 mg and heated in a thermogravimetric analyzer (TGA/DSC3+ HT, Mettler-Toledo Inc., Schwerzenbach, Switzerland) from 30 to 700 °C at a heating rate of 10 °C/min under nitrogen gas flow at 50 mL/min to measure the initial degradation temperature (T_d_) and residue.

### 4.10. Scanning Electron Microscope (SEM)

The surface morphology of CMS/CS/PT gel films with different concentrations of honey bee brood protein were imaged on an LV scanning electron microscope (JSM 5910 LV, Tokyo, Japan) operating at an accelerating voltage of 15 kV. The carbon adhesive was employed to mount the dried samples on aluminum stubs. A vacuum sputter coater (GSL-1100X-SPC12-LD, MTI, California, USA) was used to coat the samples with gold for 60 s, resulting in a 20-nanometer thickness. Each image was captured at a magnification of 1000×.

### 4.11. Statistical Analysis

The experimental data were analyzed via SPSS Statistics version 21.0 software (IBM Co., New York, NY. USA). The results are displayed as the average ± standard deviation. Duncan’s multiple range test was used throughout this study to detect significant differences among average values, with a significance level of 0.05.

## Figures and Tables

**Table 1:** Amino acid composition of honey bee brood protein.

Amino Acid	Content (mg/g Dry Basis)
Essential amino acid	
Threonine	0.08 ± 0.01
Valine	0.39 ± 0.01
Isoleucine	0.08 ± 0.01
Leucine	0.30 ± 0.02
Methionine	0.06 ± 0.01
Phenylalanine	0.37 ± 0.01
Lysine	1.01 ± 0.06
Histidine	0.58 ± 0.02
Nonessential amino acid	
Aspartic acid	0.05 ± 0.02
Serine	0.02 ± 0.01
Glutamic acid	0.72 ± 0.04
Proline	1.51 ± 0.16
Glycine	0.22 ± 0.01
Alanine	1.48 ± 0.07
Tyrosine	ND *
Arginine	ND *

Note: ND * means not detected.

**Table 2:** Surface color and optical properties of CMS/CS/PT gel films with different concentrations of honey bee brood protein.

Film	Surface Color					Opacity (mm^−1^)
	L*	a*	b*	∆E	YI	
CMS/CS/PT	78.19 ± 0.11 ^c^	2.85 ± 0.57 ^a^	27.28 ± 0.85 ^a^	34.44 ± 0.93 ^ab^	49.91 ± 5.74 ^ab^	3.66 ± 0.16 ^c^
CMS/CS/PT/0.025 BBP	77.83 ± 0.10 ^d^	3.13 ± 0.50 ^b^	27.92 ± 0.09 ^a^	35.19 ± 0.10 ^a^	51.30 ± 4.39 ^ab^	3.63 ± 0.27 ^c^
CMS/CS/PT/0.05 BBP	79.02 ± 0.35 ^b^	2.81 ± 0.50 ^b^	27.04 ± 0.34 ^a^	33.80 ± 0.54 ^b^	48.97 ± 6.76 ^ab^	4.00 ± 0.07 ^b^
CMS/CS/PT/0.1 BBP	79.57 ± 0.82 ^ab^	2.23 ± 0.32 ^c^	25.40 ± 0.81 ^b^	32.07 ± 0.95 ^c^	45.63 ± 3.65 ^b^	3.24 ± 0.08 ^d^
CMS/CS/PT/0.2 BBP	80.05 ± 0.05 ^a^	2.13 ± 0.31 ^d^	22.47 ± 0.30 ^d^	29.36 ± 0.24 ^d^	40.18 ± 6.33 ^b^	3.53 ± 0.03 ^c^
CMS/CS/PT/0.4 BBP	79.99 ± 0.30 ^a^	2.56 ± 0.52 ^d^	23.67 ± 0.78 ^c^	30.42 ± 0.84 ^d^	42.36 ± 7.28 ^b^	4.22 ± 0.10 ^a^

All data represent mean ± standard deviation; the different superscript letters (a–d) in the same column indicate a significant difference (*p* < 0.05).

**Table 3:** Mechanical properties of CMS/CS/PT gel films with different concentrations of honey bee brood protein.

Film	Thickness (mm) ^NS^	Tensile Strength (MPa)	Tensile Strength After 24 h Water Absorption (MPa)	Elongation at Break (%)	Elongation at Break After 24 h Water Absorption (%)	Young’s Modulus (MPa)
CMS/CS/PT	0.11 ± 0.01	7.95 ± 0.19 ^ab^	0.66 ± 0.07 ^c^	34.90 ± 0.33 ^a^	12.81 ± 0.53 ^c^	65.70 ± 1.28 ^e^
CMS/CS/PT/0.025 BBP	0.11 ± 0.01	6.72 ± 0.26 ^c^	0.33 ± 0.02 ^d^	25.55 ± 0.21 ^d^	7.68 ± 0.50 ^d^	105.54 ± 1.70 ^a^
CMS/CS/PT/0.05 BBP	0.11 ± 0.01	6.74 ± 0.19 ^c^	0.34 ± 0.01 ^d^	29.19 ± 0.18 ^c^	8.38 ± 0.93 ^d^	47.55 ± 1.31 ^f^
CMS/CS/PT/0.1 BBP	0.11 ± 0.01	7.73 ± 0.09 ^b^	0.35 ± 0.01 ^d^	32.23 ± 0.15 ^b^	15.45 ± 0.87 ^a^	97.84 ± 1.02 ^b^
CMS/CS/PT/0.2 BBP	0.11 ± 0.01	5.64 ± 0.22 ^de^	1.02 ± 0.03 ^b^	20.37 ± 0.13 ^e^	14.85 ± 0.24 ^b^	83.75 ± 1.16 ^d^
CMS/CS/PT/0.4 BBP	0.11 ± 0.01	5.16 ± 0.27 ^e^	2.52 ± 0.01 ^a^	17.96 ± 0.09 ^f^	14.25 ± 0.32 ^b^	88.46 ± 1.12 ^c^

All data represent mean ± standard deviation; the different superscript letters (a–f) in the same column indicate a significant difference (*p* < 0.05). NS represents no significant difference (*p* < 0.05) in the same column.

**Table 4:** Hydrophobic characteristics and barrier properties of CMS/CS/PT film with different concentrations of honey bee brood protein.

Film	Moisture Content (%) ^NS^	Contact Angle (°)	Water Absorption (%)	Thickness Swelling (%)	Water Solubility (%)	WVP × 10^−6^ (g m^−1^ d^−1^ Pa^−1^)
CMS/CS/PT (Control)	7.75 ± 0.13	76.18 ± 0.50 ^b^	288.11 ± 2.70 ^d^	117.52 ± 1.38 ^d^	35.63 ± 0.83 ^d^	4.50 ± 0.14 ^a^
CMS/CS/PT/0.025 BBP	7.38 ± 0.57	65.86 ± 0.23 ^d^	327.74 ± 9.50 ^b^	243.32 ± 1.81 ^b^	58.54 ± 0.56 ^a^	4.33 ± 0.28 ^ab^
CMS/CS/PT/0.05 BBP	7.46 ± 0.39	73.68 ± 0.45 ^c^	317.14 ± 3.46 ^c^	227.12 ± 1.36 ^c^	42.86 ± 0.43 ^b^	4.22 ± 0.29 ^ab^
CMS/CS/PT/0.1 BBP	6.92 ± 0.60	83.68 ± 0.48 ^a^	369.01 ± 4.43 ^a^	248.75 ± 1.65 ^a^	29.16 ± 0.11 ^e^	3.93 ± 0.15 ^b^
CMS/CS/PT/0.2 BBP	6.93 ± 0.42	55.76 ± 0.52 ^e^	87.47 ± 4.77 ^ef^	34.33 ± 1.33 ^e^	40.53 ± 0.33 ^c^	2.20 ± 0.36 ^c^
CMS/CS/PT/0.4 BBP	6.96 ± 0.60	51.86 ± 0.48 ^f^	81.39 ± 1.73 ^f^	21.06 ± 1.87 ^f^	36.20 ± 0.58 ^d^	2.11 ± 0.12 ^c^

All data represent mean ± standard deviation; the different superscript letters (a–f) in the same column indicate a significant difference (*p* < 0.05). NS represents no significant difference (*p* < 0.05) in the same column.

## Data Availability

All data and materials are available upon request from the corresponding author. The data are not publicly available due to ongoing research using some of the data.
